# Can artificial intelligence pass the test? Evaluating chatbot scores on pediatric gastroenterology board‐style questions

**DOI:** 10.1002/jpr3.70178

**Published:** 2026-04-07

**Authors:** Zvi Weizman

**Affiliations:** ^1^ Division of Pediatrics Faculty of Health Sciences Ben‐Gurion University of the Negev Beer‐Sheva Israel

Dear Editor,

I read with interest the study by Roberts et al.^1^ I identify several issues that warrant consideration. Loss of media files in text‐only transfer represents a fundamental limitation. Clinical gastroenterology relies heavily on visual interpretation, including endoscopic images, radiographs, and other imaging modalities. The authors acknowledge that 46 questions (21%) contained media files that were not transmitted to chatbots. Testing Artificial Intelligence on incomplete clinical vignettes does not accurately reflect the multimodal nature of board examinations. Modern Large Language Models (LLMs) with native vision capabilities, such as GPT‐4V, Gemini Pro Vision, and Claude 3, were available at the time of the study and would have provided a more valid assessment.^2^, ^3^

Another issue is the model selection. They evaluated GPT‐3.5, GPT‐4o, and Copilot, but omitted other major models, including Claude, Gemini, and specialized medical LLMs such as Med‐PaLM 2, which have demonstrated impressive performance on medical licensing examinations.^4^

The prompting methodology was minimal. The authors used a simple instruction: “Please answer the following questions.” Research has demonstrated that prompting techniques significantly influence LLM performance on medical reasoning tasks. Thoughtful structured prompts have been shown to significantly improve accuracy.^5^, ^6^

Additionally, each question was tested only once per model. LLMs exhibit variable outputs for identical inputs. Best practices in LLM evaluation recommend multiple runs to establish reliable estimates.^4^ Single‐run testing introduces unnecessary measurement uncertainty.

Furthermore, the domain‐level analysis suffers from limited statistical power. Several categories contained only 2–6 questions, making performance comparisons unreliable. Using such small samples is likely to lead to overgeneralization.

## CONFLICT OF INTEREST STATEMENT

The author declares no conflict of interest.
